# Comparison of corneal measurements using two different Scheimpflug analyzers in Sirius and Pentacam devices

**DOI:** 10.1038/s41598-023-44133-3

**Published:** 2023-10-07

**Authors:** Parisa Abdi, Mehrnaz Atighehchian, Amir Reza Farsiani

**Affiliations:** 1 Farabi Eye Hospital, Tehran University of Medical Sciences, Tehran, Iran; 2 Department of Ophthalmology, School of Medicine, Zanjan University of Medical sciences, Zanjan, Iran

**Keywords:** Medical research, Optics and photonics

## Abstract

The aim was to compare measurements of anterior segment biometry parameters using two Scheimpflug tomographies, Pentacam and Sirius to assess the agreement. Prospective cross-sectional observational study. A total of 60 eyes of 30 healthy subjects were included and evaluated with Pentacam followed by Sirius imaging. Corneal indices were performed with two modalities in both eyes including; apical corneal thickness (ACT), corneal thickness at pupil site(PCT), thinnest corneal thickness (TCT), anterior chamber depth (ACD), chamber angle, chamber volume, cornea volume, mean front keratometry (FKm), the radius of corneal curvature at the anterior and posterior surface in steep and flat meridian, anterior astigmatism values, pupil diameter, and horizontal corneal diameter. The Bland–Altman graph and ICC (intra-class correlation were used to establish an agreement and present the similarity of the findings. Most parameters showed perfect agreement. In both devices, the ICC was more than 0.91 in all measurements except for ACD (ICC = 0.820), cylinder axis (ICC = 0.520), TCT(ICC = 0.881), ACT(ICC = 0.672), PCT (ICC = 0.882), chamber angle (ICC = 0.362), pupil diameter(ICC = 0.137). Pentacam yielded higher values that were significant in five parameters including 3.47 μm for TCT, 4.29 µm for PCT, 10.03 mm^3^ for chamber volume,2.67 mm^3^ for cornea volume, and 1.49 mm for pupil diameter but there was only a statistically significant difference in cornea volume and pupil diameter (p-value < 0.001). However, in Pentacam only the chamber angle value was 6.44 mm^3^ lower than Sirius, with a statistically significant difference (p-value < 0.001). Although these two devices had some statistically different results, it seems that they have a good agreement and correlation in most parameters.

## Introduction

The importance of precise measurement of corneal thickness, corneal curvature, and ACD has increased recently because of the development in corneal refractive surgeries and phakic intraocular lens (PIOL) implantation^1^.

Also, these measurements are important for managing corneal pathologies such as ectasia to evaluate the progression of corneal thinning and measuring curvature values^2,3^.

Many types of instruments have been used to evaluate anterior segment parameters, but there is no gold standard to check the accuracy of parameters. So, it is essential to know the differences between the values of these parameters measured by different techniques of corneal topography devices.

Scheimpflug photographic devices have become available since 2013^4,5^. Also, newer modalities with rotating Scheimpflug cameras that are combined with a Placido-based corneal topography system have been introduced. Two of the most common devices used for anterior segment measurements are the Pentacam (Pentacam HR, Oculus, Wetzlar, Germany) and the Sirius (CSO, Florence, Italy). The Pentacam system uses a single Scheimpflug camera and the Sirius combines a Scheimpflug camera with a Placido disc corneal topographer to better analyze the corneal curvature^4–6^. These non-contact devices can evaluate anterior segment data such as the total corneal dioptric power of the anterior and posterior corneal surfaces, corneal pachymetry, ACD, and volume^1,7^.

Although few studies have assessed the agreement of the Pentacam measurements with Sirius, it is still necessary to determine whether the results of these devices are comparable and if they can be used interchangeably^8,9^. So, the purpose of the present study is to evaluate the agreement of two Scheimpflug tomographies (Pentacam, Sirius) in measuring the corneal and anterior chamber parameters in the healthy cornea.

## Materials and methods

This prospective study adhered to the tenets of the Declaration of Helsinki and the protocol of the study was approved by the Ethics Committee of Tehran University of Medical Sciences, Tehran, Iran. All participants were informed about the study goals and informed consent was obtained.

Participants included subjects who were healthy and who were scheduled for refractive surgery at the Department of Ophthalmology, Farabi Eye Hospital, Tehran University, Iran. The exclusion criteria were any corneal pathology, eyelid abnormalities, history of intraocular surgery, patients with recent contact lens wear (rigid contact lens for more than 4 weeks and soft contact lens for more than 2 weeks), and ectatic disorders.

A total of 60 eyes of 30 healthy subjects were included. To compare the two sets considering bilateral eyes, a linear mixed model was used. The graphical agreement assessment with the Bland–Altman graph was used to establish an agreement between devices. We used ICC (intra-class correlation) to present the similarity of the findings.

After providing a detailed medical history, all participants underwent complete ophthalmic examinations; Then subjects were evaluated with Pentacam followed by Sirius imaging that was taken in a non-dilated pupil in proper lighting conditions. Subjects were instructed to blink completely before each imaging acquisition. All tests were carried out on the same day, by the same trained operator, and the image quality scores were confirmed to be "acceptable". For each eye, all measurements were taken at least three times, and the best-quality image was used for statistical analysis.

### Sirius

The Sirius system combines a 360° rotating Scheimpflug camera along with a small-angle Placido disk-based corneal topographer with 22 rings acquiring 25 radial sections of the cornea. The 22 rings provide height, slope, and curvature data, that are obtained by an arc-step method with conic curves. Details for the anterior cornea are collated from data from both the Placido disk and Scheimpflug images. Data for the posterior corneal surface, anterior lens, and iris is obtained from Scheimpflug images. The system can measure 35,632 points from the anterior cornea and 30,000 points from the posterior corneal surface and display sagittal and tangential corneal curvature for anterior and posterior surfaces. The pachymetry map is then reconstructed using the data from both corneal surfaces within 5 to 6 s of acquisition time^8,10^.

### Pentacam

The Pentacam uses a rotating Scheimpflug camera (360 degrees) and a monochromatic slit-light source (blue light-emitting diode at 475 nm) that rotate around the optical axes of the eye to calculate three-dimensional (3-D) anterior segment values. The system has 2 scanning modes. One is a 3-D scan that takes 50 images in 2 s, and the other one is a 3-D high-resolution cornea-scanning that takes 50 images in 1 s. More than 25,000 elevation points are used to give a three-dimensional representation of the cornea^4,8^.

We selected the following parameters in Table 1 to assess the agreement between Sirius and Pentacam.

**Table 1:** Data collected from normal patients.

Parameter	Pentacam	Sirius	Difference	95% CI		P-value^14^
				Lower	Upper	
ACD^1^ (mm)^2^	3.34 ± 0.34	3.33 ± 0.33	0.01 ± 0.2	−0.04	0.06	0.851
Corneal cylinder(D)^3^	−1.29 ± 0.79	−1.1 ± 0.77	−0.19 ± 0.24	−0.25	−0.13	0.001
FKm^4^(D)	43.44 ± 1.16	43.21 ± 1.13	0.23 ± 0.24	0.17	0.29	0.276
TCT^5^(µm)^6^	529.03 ± 24.67	525.56 ± 25.94	3.47 ± 12.34	0.26	6.69	0.458
ACT^7^(µm)	532.47 ± 24.74	545.79 ± 39.55	−13.31 ± 26.71	−20.27	−6.35	0.031
PCT^8^ (µm)	532.02 ± 24.51	527.73 ± 26.1	4.29 ± 12.29	1.09	7.49	0.359
Chamber volume (mm^3^)	209.34 ± 39.81	199.31 ± 33.3	10.03 ± 18.43	5.23	14.83	0.141
Chamber angle (°)^9^	40.93 ± 6.58	47.38 ± 5.86	−6.44 ± 7.04	−8.28	−4.61	0.001
Cornea volume (mm^3^)	59.05 ± 2.74	56.38 ± 2.62	2.67 ± 1.12	2.38	2.96	0.001
FRf^10^ (mm)	7.89 ± 0.25	7.81 ± 0.24	0.08 ± 0.05	0.07	0.09	0.083
FRs^11^( mm)	7.66 ± 0.19	7.6 ± 0.18	0.05 ± 0.05	0.04	0.07	0.123
BRf ^12^ (mm)	6.62 ± 0.25	6.64 ± 0.25	−0.01 ± 0.06	−0.03	0.01	0.797
BRs ^13^ ( mm)	6.27 ± 0.21	6.33 ± 0.21	−0.05 ± 0.05	−0.07	−0.04	0.172
Pupil diameter (mm)	3.42 ± 1.21	1.93 ± 0.3	1.49 ± 1.16	1.19	1.8	0.001
Horizontal corneal diameter(mm)	12.03 ± 0.41	12.26 ± 0.39	−0.23 ± 0.12	−0.26	−0.2	0.002

(1)  Anterior chamber depth, (2)  millimeter, (3)  Diopter, (4)  mean front keratometry, (5)  thinnest corneal thickness, (6)  micrometer, (7)  apex corneal thickness, (8)  pupil corneal thickness, (9)  degree, (10)  flat radius of front surface, (11)  steep radius of front surface, (12)  flat radius of back surface, (13)  steep radius of back surface, (14)  based on linear mixed model.

Placido disc topographer in the Sirius provides more reliable anterior corneal curvature measurements and measures geometrical corneal slope values that are converted into axial curvature values. Sirius calculates the keratometric diopters by averaging the axial curvature from the fourth to the eighth Placido rings. Pentacam measures geometrical height (elevation) values, which are converted into values of axial (sagittal) or instantaneous (tangential) curvature and given in mm. In both devices, these values are converted from radius to diopters using the keratometric index of 1.3375^4,8^. To assess keratometric data of the anterior corneal surface, Pentacam uses only the Scheimpflug images, whereas Sirius uses the Placido disk^4^. Anterior chamber depth (ACD) value is the mean of measurement of Scheimpflug scans in both devices^5,8^.

### Statistical analysis

To present data, we used mean, standard deviation, median, and range. To compare the two sets considering the bilateral eyes we used a linear mixed model. Also, in the construction of limits of agreement(LoA) considering the possible correlation and agreement of findings we utilized the linear mixed-effects model analysis in the mentioned evaluations (Parker RA, Scott C, Inácio V, Stevens NT. Using multiple agreement methods for continuous repeated measures data: a tutorial for practitioners. BMC Med Res Methodol. 2020; 20:154). We used ICC (intraclass correlation) and r (Pearson Correlation Coefficient relation) to present the similarity of the findings. Also, to present the correction formula to transform values from Sirus to Pentacam we used linear regression analysis and the following formula. Pentacam _value = Beta0 + Beta1 * Sirus_value. The correction ability of these formulae is presented by Pearson correlation coefficients.

## Results

Sixty eyes of 30 subjects (13 males,17 females) were analyzed. The mean age was 29.23 years old.

### Agreement of thinnest corneal thickness (TCT) and apex corneal thickness (ACT) measurements

TCT was measured using Pentacam and Sirius with mean ± SD of 529.03 ± 24.67, and 525.56 ± 25.94 μm, respectively. TCT measurement with Pentacam was thicker than those analyzed with Sirius. The difference was 3.47 ± 12.34 μm (95% CI: 0.26 to 6.69) but was not significant (p-value: 0.458). The correction formula to transform values from Sirius to Pentacam was Beta0 = 88.101 and Beta1 = 0.839. The 95% limit of agreement (LoA) between Pentacam and Sirius in the measurement of the TCT was −20.71 and 27.65 μm. There were significant correlations between Pentacam and Sirius (r = 0.882; ICC: 0.881) in the measurement of the TCT.

The mean ACT measurement was 532.47 ± 24.74 μm with the Pentacam and 545.79 ± 39.55 μm with Sirius. ACT measurement with Pentacam was thinner than those evaluated with Sirius. The difference was −13.31 ± 26.71 μm (95% CI: −20.27 to −6.35), which was significant (p-value: 0.031). The correction formula to transform values from Sirius to Pentacam was Beta0 = 277.281 and Beta1 = 0468.

The 95% LoA of Pentacam with Sirius in the measurement of the ACT was − 65.66 and + 39.04 mm. There were no significant correlations between Pentacam and Sirius (r = 0.774; ICC: 0.672) in the measurement of the ACT.

### Agreement of ACD measurements

ACD was measured using Pentacam and Sirius with a mean of 3.34 ± 0.34 mm, and 3.33 ± 0.33 mm, respectively. The difference was 0.01 ± 0.2 mm (95% CI: −0.04 to 0.06) which was not significant (p-value: 0.851). The correction formula to transform values from Sirius to Pentacam was Beta0 = 0.554 and Beta1 = 0.837.

The 95% LoA of Pentacam with Sirius in the measurement of the ACD was − 0.382 to + 0.402 mm. There were good correlations between Pentacam and Sirius (r = 0.821; ICC: 0.820) for measuring the ACD.

### Agreement of mean front keratometry (FKm) measurements

FKm was measured using Pentacam, and Sirius with mean 43.44 ± 1.16 D and 43.21 ± 1.13 D, respectively. The 95% LoA of Pentacam with Sirius in the measurement of the Km was −0.24 to 0.70 D. There were excellent correlations between Pentacam and Sirius (r = 0.978) in the measurement of the FKm. The ICC was 0.978 with 95% CI: 0.963 to 0.987.

### Agreement of other indices

Tables 1 and 2 summarize all data Measurements and LoA between Pentacam, and Sirius.

**Table 2:** Data measurements and limits of agreement in Pentacam, and Sirius.

Parameter	ICC	95% CI		95% LoA^14^		R^15^	Beta0^16^	Beta1^17^
		Lower	Upper	Lower	Upper			
ACD^1^(mm)^2^	0.820	0.715	0.889	−0.382	0.402	0.821	0.554	0.837
Corneal cylinder(D)^3^	0.953	0.922	0.972	−0.6604	0.2804	0.953	−0.212	0.979
FKm^4^(D)	0.978	0.963	0.987	−0.2404	0.7004	0.978	0.257	0.999
TCT^5^(µm)^6^	0.881	0.808	0.928	−20.7164	27.656	0.882	88.101	0.839
ACT^7^(µm)	0.672	0.504	0.791	−65.6616	39.042	0.774	277.281	0.468
PCT^8^(µm)	0.882	0.810	0.829	−19.7984	28.378	0.884	94.046	0.830
Chamber volume (mm^3^)	0.874	0.797	0.923	−26.0928	46.153	0.888	−2.266	1.062
Chamber angle (°)^9^	0.362	0.118	0.564	−20.2384	7.3584	0.364	21.568	0.409
Cornea volume (mm^3^)	0.913	0.858	0.947	0.4748	4.8652	0.914	5.249	0.954
FRf^10^ (mm)	0.982	0.969	0.989	−0.018	0.178	0.983	−0.205	1.036
FRs^11^ (mm)	0.968	0.946	0.981	−0.048	0.148	0.968	0.256	0.973
BRf^12^ (mm)	0.966	0.944	0.980	−0.1276	0.1076	0.968	0.162	0.974
BRs^13^ (mm)	0.969	0.948	0.981	−0.148	0.048	0.969	0.087	0.978
Pupil diameter (mm)	0.137	−1.22	0.378	−0.7836	3.7636	0.296	1.097	1.206
Horizontal corneal (mm)	0.952	0.920	0.971	−0.4652	0.0052	0.953	−0.091	0.989

(1)  Anterior chamber depth, (2)  millimeter, (3)  Diopter, (4)  mean front keratometry, (5)  thinnest corneal thickness, (6)  micrometer, (7)  apex corneal thickness, (8)  pupil corneal thickness, (9)  degree, (10)  flat radius of front surface, (11)  steep radius of front surface, (12)  flat radius of back surface, (13)  steep radius of back surface, (14)  limit of agreement based on linear mixed model analysis, (15)  r: Pearson correlation coefficient, (16)  Beta0: intercept of correction formula based on linear regression, (17)  Beta1: the correction coefficient based on linear regression.

**Figure 1:**
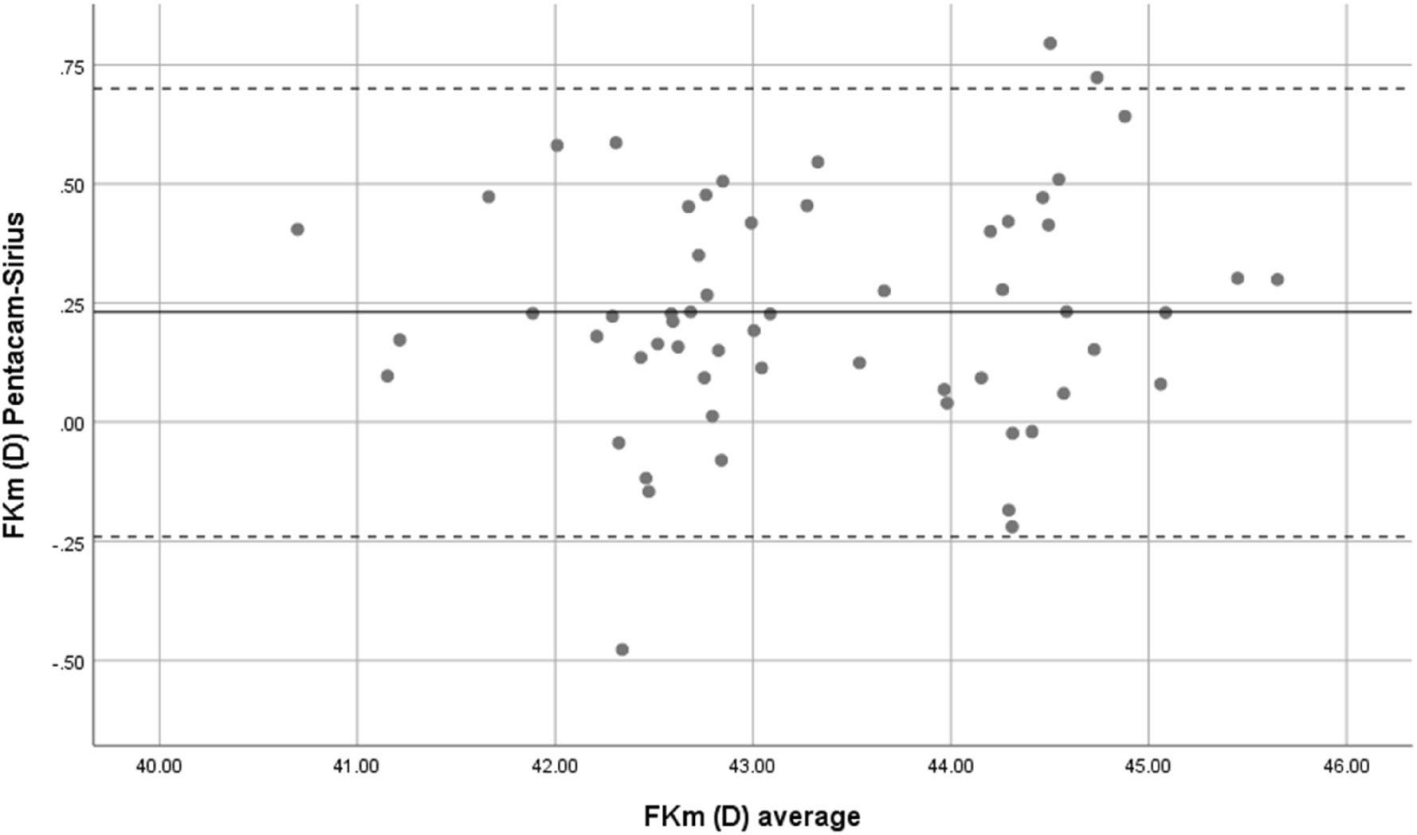
Bland–Altman plot shows the correlation agreement between mean front keratometry (FKm) measurements from Sirius and Pentacam in normal subjects.

**Figure 2:**
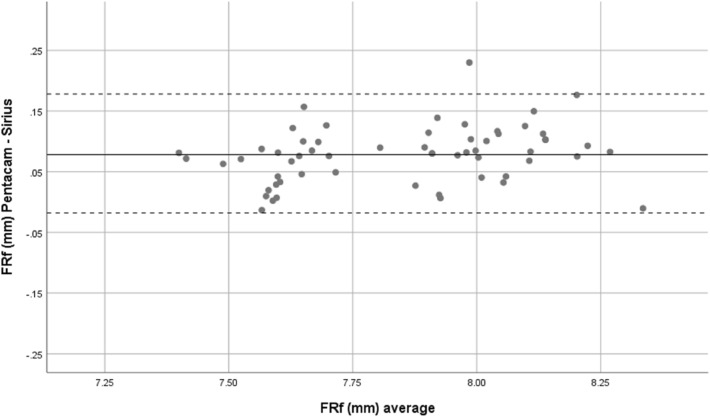
Bland–Altman plot shows the correlation agreement between radius of corneal curvature at the front surface in flat meridian (FRf) from Sirius and Pentacam.

**Figure 3:**
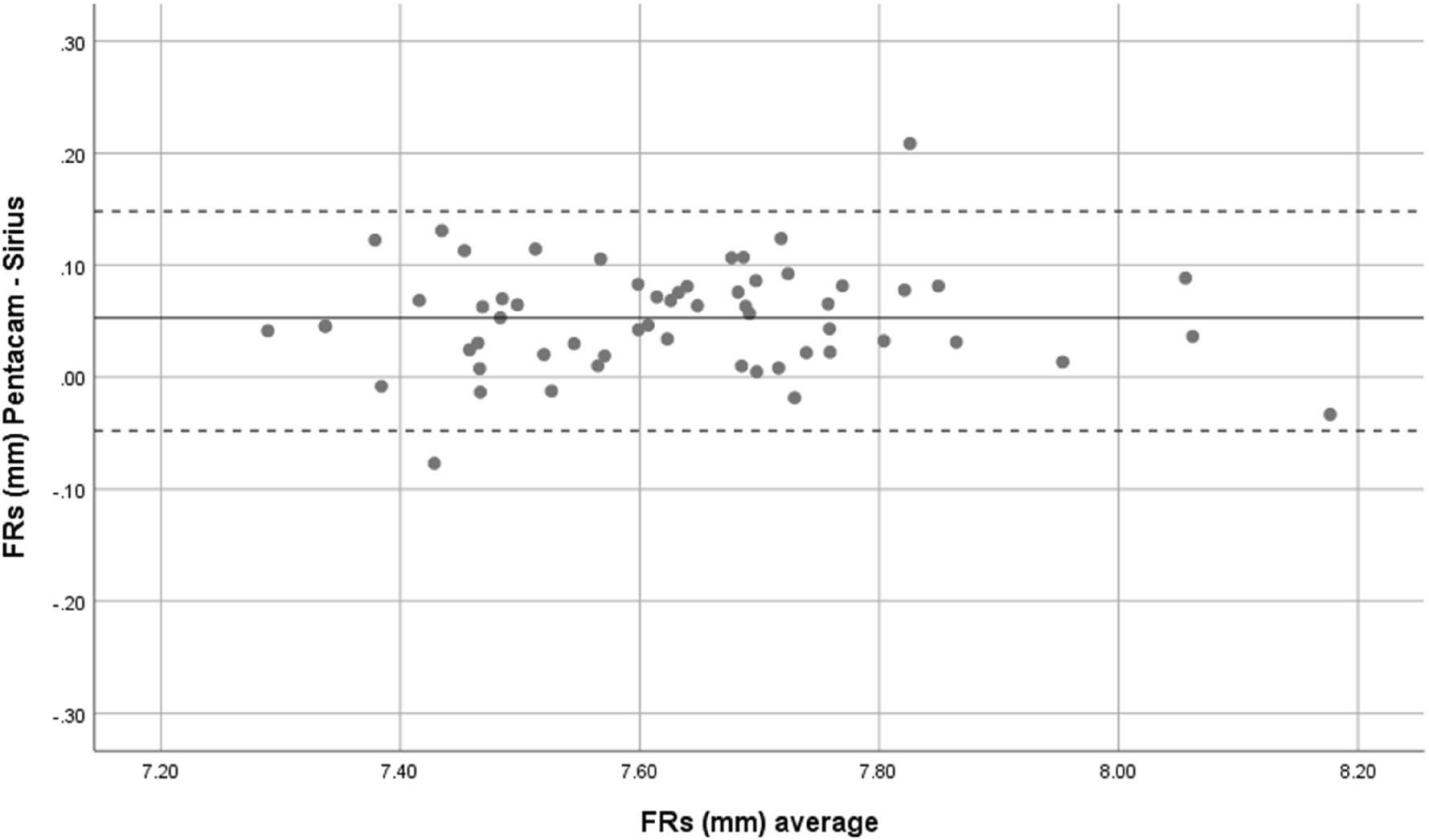
Bland–Altman plot shows the correlation agreement between radius of corneal curvature at the front surface in steep meridian (FRs) from Sirius and Pentacam.

**Figure 4:**
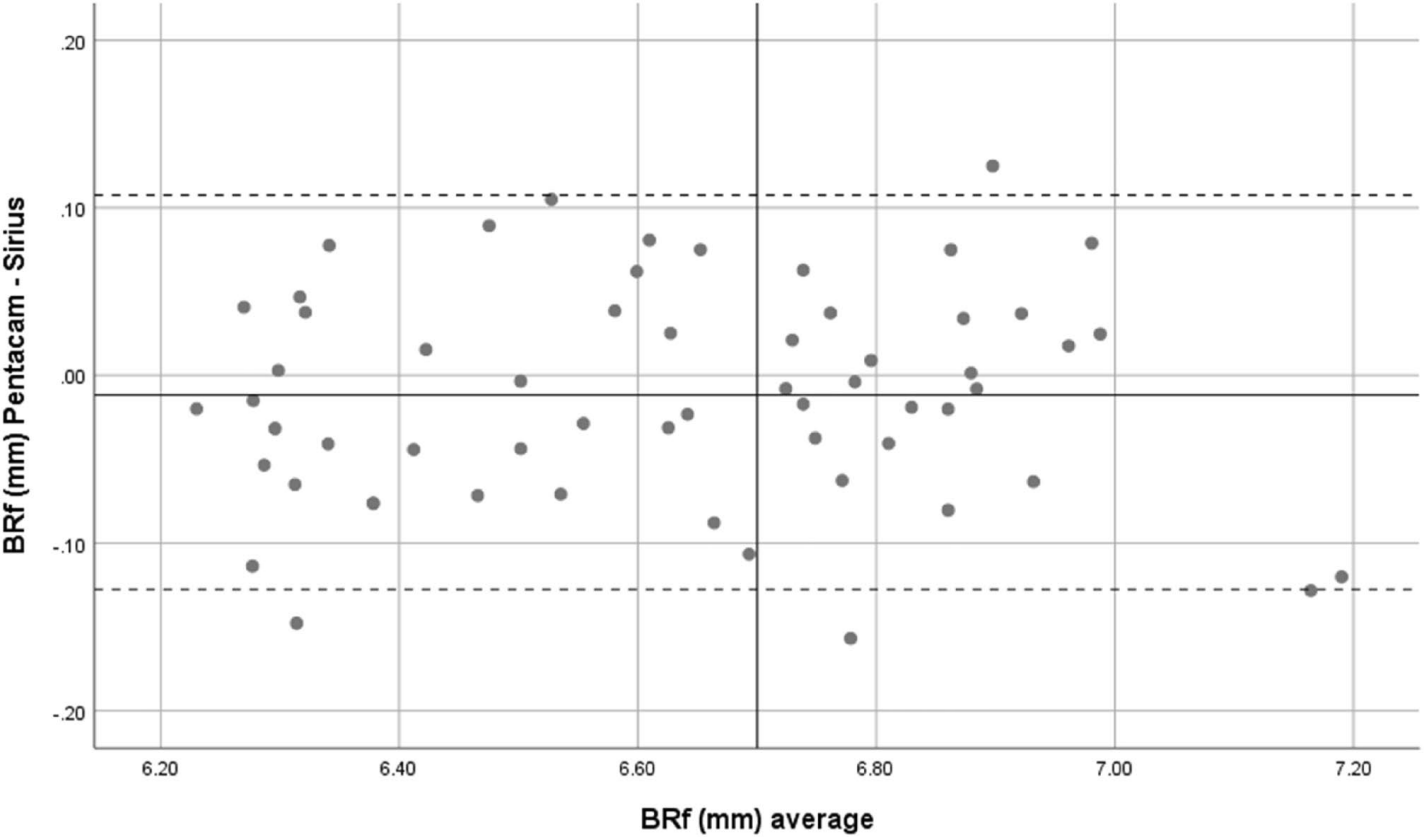
Bland–Altman plot shows the agreement between radius of corneal curvature at the back surface in flat meridian (BRf) from Sirius and Pentacam.

**Figure 5:**
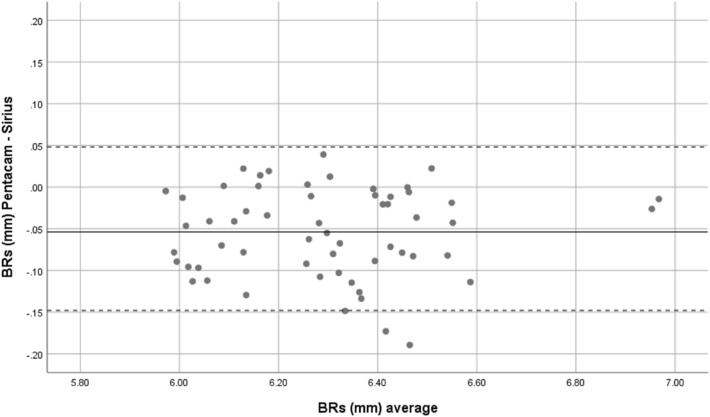
Bland–Altman plot shows the agreement between radius of corneal curvature at the back surface in steep meridian (BRs) from Sirius and Pentacam.

Overall, mean front corneal keratometry and front and back corneal curvature indices showed perfect and strong agreement. Bland–Altman plots were used to show the difference between the two devices. Therefore, both Sirius and Pentacam showed good agreement in corneal power indices, Figs. 1, 2, 3, 4 and 5.

The number of corneal cylinders had excellent agreement. Moreover, ACD and corneal and chamber volume had moderate to strong agreement. Thickness indices PCT and TCT showed perfect agreement between the two devices whereas ACT showed fair agreement. Horizontal corneal diameter showed a strong agreement; however, there was not enough agreement and a good correlation in the values of pupil diameter between the two instruments (r: 0.296, ICC: 0.137).

## Discussion

The importance of accurate corneal evaluation for corneal refractive surgery has increased recently. Both Pentacam and Sirius are performed to measure anterior segment parameters from the anterior corneal surface to the posterior lens surface^2^. But these devices have different artificial intelligence and use different methods to measure various parameters. Therefore, some of the corneal measurements with these instruments cannot be considered interchangeable^4^.

While Pentacam has settings for 25 and 50 three-dimensional scans, Sirius has a 25-scan with one Placido image setting^10^. Sirius has a high-resolution placido topographer that allows for the evaluation of total corneal wavefront, as well as corneal aberrations that can help the clinician to understand the patient’s visual abnormalities. Both devices have keratoconus screening systems and help to diagnose high-risk patients before refractive surgery^2,8,11,12^.

Both devices have been used recently and patients are evaluated with one of these devices. An ophthalmologist needs to know their compatibility. To our knowledge, there are a few studies in this field of literature. However, our study has some important differences from previous studies. Other studies compared only a limited number of variables (pachymetry, Anterior chamber depth, and, keratometry index), while we considered more than ten parameters and compared more parameters between these two devices^4,9,10^. Also, some of these studies only compared the repeatability of the parameters between these devices and the agreement was not evaluated. This is different from our study^8^.

In the present study, we analyzed the agreement of 15 cornea and anterior chamber parameters between Sirius and pentacam, which has been unprecedented, until now. Also, we presented a corrected formula for converting Sirius and pentacam data results to correct the difference indices. This correction formula has not been presented in any other studies.

The present study was designed to compare the TCT, PCT, ACT, ACD, anterior chamber volume (ACV), chamber angle (CA), FKm, and radius of corneal curvature at the front and back surface in flat and steep meridian values(FRs, FRf, BRs, BRf) between these two Scheimpflug devices and evaluated the agreement between these parameters.

TCT and PCT are the main parameters to diagnose corneal ectasias^4,13^. The findings in this study suggest that TCT and PCT measured with Pentacam were thicker than those analyzed with Sirius (3.47, 4.29 μm). However, no statistically significant difference was observed (p > 0.05). There was a good correlation between Pentacam and Sirius in the measurement of the TCT (ICC = 0.881 r = 0.882) and PCT (ICC = 0.882, r = 0.884). On the other hand, the ACT is thicker in Sirius than Pentacam (−13.31 ± 26.71 μm). We found a significant difference (P-value: 0.031) between these two devices so there was a fair agreement(ICC = 0.672) for this index.

Accurate keratometry measurements are important in refractive surgery and for IOL power calculation^14^. Previous studies, reported good agreement between anterior corneal power values and corneal curvature determined by both Pentacam and Sirius^15^. In this study both devices showed excellent agreement for FKm Measurements (ICC = 0.978). Although the mean difference between FRs and FRf was not statistically significant, the agreement expressed by the 95% LoA values in FRs and FRf showed that these indices can be used interchangeably in clinical evaluation.

In addition, the mean difference between the two devices in BRs and BRf was not significant and the agreement revealed by the 95% LoA values was from −0.14 to 0.04 and from −0.12 to 0.10 for BRs and BRf, respectively. So, these indices can be used interchangeably.

Horizontal ACD measurement has become necessary in cataract and refractive surgery or phakic IOL implantations for accurate IOL power calculation^16,17^. Previous studies, evaluated the agreement in ACD measurements of normal eyes obtained from Orbscan, Pentacam, and Galilei. They found that the ACD measurements of Orbscan are not interchangeable with Galilei or Pentacam^18^. In our study, we found a 0.01 ± 0.2 mean difference between the Pentacam and Sirius for measuring ACD, however, it was not statistically significant and the agreement by the 95% LoA values suggested that ACD values can be used interchangeably in clinical preparation examination. Moreover, there was excellent agreement in chamber volume and corneal volume.

The accurate measurement of horizontal corneal diameter is important in refractive surgery. It is used for IOL power calculation in cataract surgery and phakic IOL implantation and the diagnosis and monitoring of different ocular diseases, such as megalo cornea, micro cornea, and, glaucoma^19^. Pentacam uses iris camera optics that can automatically calculate horizontal corneal diameter(HCD) with photographs taken of the iris^19^. The Sirius system measures the HCD as the horizontal visible iris diameter (HVID), that is the distance between the right and left iris edges of the grayscale image sampled on the horizontal meridian passing through the corneal vertex^20^. The present study showed that the horizontal corneal diameter in Sirius had a significantly higher value, about 0.23 mm, than Pentacam (p: 0.002), although there was good correlation between two devices to assess horizontal corneal diameter (ICC, r: 0.95).

The main limitation of our study is that we only enrolled healthy eyes because we planned to evaluate the agreement of these devices in normal conditions and the evaluation of ectatic patients is suggested. On the other hand, our study introduced a new correction formula to convert Sirius parameter values to Pentacam values to correct the difference between parameters so, in this way, these data are used interchangeably.

## Conclusion

Our results suggested that both Sirius and Pentacam are reliable devices for corneal and anterior segment biometry in normal corneas, but they cannot be used interchangeably for all parameters. However, many of the parameters have good agreement or can be correlated with an almost accurate coefficient. Our study supports the results of previous comparative research.

## Data Availability

All data generated or analyzed during this are included in this published article.
